# Detection of Euglycemic Diabetic Ketoacidosis During Thoracic Surgery 75 Hours After Empagliflozin Discontinuation

**DOI:** 10.7759/cureus.29974

**Published:** 2022-10-06

**Authors:** Ai Nishida, Osamu Ogawa, Hiroki Takizawa

**Affiliations:** 1 Department of Diabetes and Endocrinology, Kameda Medical Center, Kamogawa, JPN; 2 Information Management Headquarters, Kameda Medical Center, Kamogawa, JPN; 3 Department of Diabetes and Endocrinology, Kameda Medical Center, Kamogawa City, JPN; 4 Department of Metabolism and Endocrinology, Juntendo University, Tokyo, JPN

**Keywords:** blood ketone measurement, blood gas analysis, sglt2i, empagliflozin, euglycemic diabetic ketoacidosis

## Abstract

Euglycemic diabetic ketoacidosis (eDKA) has been increasingly reported as an adverse event of sodium-glucose cotransporter 2 inhibitors (SGLT2i), and the accompanying information on the drug recommends discontinuation three days prior to scheduled surgery. We present a case of a 50-year-old woman who developed eDKA during surgery for a metastatic lung tumor 75 hours after discontinuing SGLT2i. In this case, the onset of eDKA was detected using intraoperative blood gas analysis and urinary ketone measurements. Hence, perioperative eDKA can occur even after three or more days of SGLT2i withdrawal.

## Introduction

Sodium-glucose cotransporter 2 inhibitors (SGLT2i) are drugs for diabetes treatment that promote urinary glucose excretion. They possess cardioprotective and renoprotective properties. Therefore, they prevent any macro- or microvascular diabetic complications [1,2]. Diabetic ketoacidosis (DKA) is a serious side effect of using SGLT2i. A series of reports of euglycemic DKA (eDKA) with blood glucose levels below 250 mg/dL during SGLT2i use have been published [3]. Perioperative eDKA has also been reported when using SGLT2i [4]. However, there are few reports of eDKA that were diagnosed intraoperatively [5]. We report a case of intraoperatively detected eDKA 75 hours after empagliflozin discontinuation.

This article was previously presented as a meeting abstract at the 664th Kanto Regional Meeting of the Japanese Society of Internal Medicine on November 14, 2020.

## Case presentation

The patient was a 50-year-old woman. She was diagnosed with type 2 diabetes at age 36. Prior to her admission, she was on a drug regimen of vildagliptin 100 mg/day, metformin 1,000 mg/day, miglitol 225 mg/day, empagliflozin 10 mg/day, and multiple insulin injection therapy (insulin glulisine and insulin glargine) with a total daily dose (TDD) of 36 units. She had been prescribed empagliflozin four years prior to admission. No urinary ketones were noted during the outpatient course. Her only other medical history was a total hysterectomy for atypical leiomyosarcoma at age 46. At the time of hysterectomy, the patient was taking empagliflozin until the day before surgery. Oral intake was adequate, and preoperative urine ketones were negative. After stopping empagliflozin on the day of surgery, her urine ketone body test was positive on the third postoperative day with adequate oral intake, but there was no diagnosis of ketoacidosis. Two months prior to her admission, she was diagnosed with a metastatic lung tumor and was scheduled for surgery. One week prior to surgery, she developed chest pain on the right side along with dyspnea. She was admitted to the hospital and diagnosed with a ruptured tumor. On admission, she had no gastrointestinal symptoms. She had generalized fatigue but was conscious. Her body height was 154.9 cm, body weight was 87.7 kg, and BMI was 36.5 kg/m^2^. Her physical examination revealed no abnormal heart sounds, but the breath sounds were better heard in the left lung than in the right lung. No dry skin or skin rash was observed. Her blood test data on admission are shown in Table 1. She had no evidence of acidemia at the time of admission. After admission, she was able to eat 70% of a 1600 kcal/day diet until the day before surgery. In addition, all oral hypoglycemic medications were discontinued, and her blood glucose level remained below 200 mg/dL. The patient's blood glucose levels were managed with a sliding scale insulin regimen. Her last preoperative insulin dose was 2 units of regular insulin subcutaneously administered 36 hours before the surgery. Her last empagliflozin dose was 72 hours before surgery. On the fourth day of hospitalization, after 15 hours of overnight fasting, the patient underwent right upper lobectomy, thoracic hematoma removal, and axillary lymph node dissection. The pathology result was leiomyosarcoma.

**Table 1:** Laboratory data at admission Na, sodium; K, potassium; Cl, chloride; BUN, blood urea nitrogen; AST, aspartate aminotransferase; ALT, alanine aminotransferase; Alb, albumin; CRP, C-reactive protein; WBC, white blood cell count; RBC, red blood cell count; Hb, hemoglobin; Plt, platelet; BGA, blood gas data analysis; PaCO_2_, partial pressure of carbon dioxide; PaO_2_, partial pressure of oxide; HCO_3-_, bicarbonate ion; BE, base excess

Data		Unit
Na	141	mEq/L
K	4.5	mEq/L
CL	110	mEq/L
BUN	13	mg/dL
Creatinine	0.39	mEq/L
AST	12	IU/L
ALT	19	IU/L
Alb	3.3	g/dL
CRP	2.13	mg/dL
WBC	179	×10^2^/µL
RBC	422	×10^4^/µL
Hb	12.8	g/dL
Plt	26.8	×10^4^/µL
Hemoglobin A1c	9.2	%
BGA		
pH	7.372	
PCO_2_	35.2	Torr
PO_2_	68.1	Torr
HCO_3_-	20	mmol/L
BE	-4.5	mmol/L
Glucose	229	mg/dL
Lactate	1.3	mmol/L

She was started on an intravenous infusion of sodium L-lactate Ringer's solution without insulin and carbohydrate from 2 hours before surgery. She had an intraoperative blood glucose level of 159 mg/dL but showed metabolic acidosis. When the metabolic acidosis did not improve, a urinary ketone test was performed, which showed a value of 3+.

She was diagnosed with eDKA and started on continuous intravenous insulin. Insulin infusion was continued postoperatively. The course of blood gas analysis (BGA) is shown in Table 2. Four and a half hours after the surgery, the acidosis improved to reach pH 7.367. She had no gastrointestinal symptoms after waking up from anesthesia.

**Table 2:** Laboratory testingperioperative phase and clinical course PaCO_2_, partial pressure of carbon dioxide; PaO_2_, partial pressure of oxide; HCO_3-_, bicarbonate ion; BE, base excess; Na, sodium; K, potassium; Cl, chloride; ICU, intensive care unit; h, hours

								At the end of surgery	ICU admission				
	Surgery-3 h	Surgery start +1 h	3 h	4 h	5 h	6 h	8 h	7 h	9 h	10 h	11 h	12 h	24 h
pH	7.303	7.139	7.113	7.112	7.091	7.132	7.178	7.192	7.216	7.266	7.316	7.349	7.44
PaCO_2_ (mmHg)	33.8	48.4	47.5	47.9	52.3	41.9	36.7	38.1	37.8	33.2	32.5	31.8	29.3
PaO_2_ (mmHg)	41.9	91.4	119.2	128	118.4	396.8	409.4	412.6	151	162	89.2	89.7	77.4
HCO_3_^-^ (mmol/L)	16.4	16.1	14.9	15	15.5	13.7	13.3	14.3	15	14.8	16.2	17.1	19.5
BE (mmol/L)	-9	-12.6	-14.1	-14	-13.8	-14.6	-14	-12.9	-11.9	-11.2	-9	-7.7	-4
Na (mmol/L)	136	138	139	138	141	138	140	140	140	141	143	145	138
K (mmol/L)	3.6	3.8	4.3	4.5	4.4	4.5	4.4	4.1	3.9	3.8	3.5	3.8	4.1
CL (mmol/L)	103	104	103	104	105	105	105	106	106	106	109	107	105
Lactate (mmol/L)	0.8								1	1.2	1.2		
Glucose (mg/dL)	151	159	181	190	189	197	188		192	175	156	152	160
						Urine ketone positive							
						Start insulin infusion							

She was started on food the day after surgery, and her insulin injections were changed from intravenous to subcutaneous. She was discharged on the 14th postoperative day. Two days prior to discharge, she resumed SGLT2i, and her discharge prescription was empagliflozin 10 mg and insulin multiple injection therapy (insulin glulisine and insulin glargine) with a TDD of 38 units. Her pre-discharge testing was negative for glutamic acid decarboxylase antibodies, and her urine ketones were negative 12 days after the discharge.

## Discussion

Two clinical findings are highlighted in this study. First, intraoperative eDKA can occur even after 75 hours of empagliflozin discontinuation. Second, intraoperative measurement of ketones and BGA is useful for the early detection of eDKA. These findings are elaborated on further below.

Perioperative eDKA occurrence after 75 hours of empagliflozin discontinuation

eDKA has traditionally been reported as an adverse event of SGLT2i [6,7]. In addition, perioperative SGLT2i-related DKA has been reported [4,8-13]. There are reports of intraoperative eDKA diagnosed 28 hours after empagliflozin discontinuation [5]. It is therefore recommended that SGLT2i be discontinued three to four days prior to surgery [14,15]. The U.S. Food and Drug Administration has revised the package insert to discontinue canagliflozin, dapagliflozin, and empagliflozin three days before scheduled surgery on March 15, 2022 [16].

In this case, eDKA was diagnosed intraoperatively 75 hours after empagliflozin discontinuation. It should be noted that DKA may occur even after a sufficient withdrawal period. Several triggers of SGLT2i-related DKA have been identified [17]. According to the review, surgical stress, insufficient pre-/post-operative or reduced dose of insulin, reduced perioperative oral intake, infection, malignancy, dehydration, and decreased oral intake are some known triggers [17]. In this case, the triggers were lower insulin dosage, lower oral intake than before admission, inflammatory response, presence of malignancy, and surgical stress. Based on the above, we assume that the patient had SGLT2i-associated eDKA due to a combination of triggers, including decreased insulin dosage, decreased oral intake, inflammatory response, presence of malignancy, and surgical stress. The patient had a previous history of positive urine ketones in the perioperative period. This case could have been prone to ketosis due to some triggers even after discontinuation of SGLT2i. Therefore, eDKA may have occurred even after 75 hours of the last empagliflozin administration. Even if blood glucose levels were not high, preoperative intravenous infusion with glucose and insulin could have facilitated the prevention of SGLT2i-related eDKA.

It is useful to measure ketones and BGA during surgery for early detection of intraoperative eDKA

Diagnosis of intraoperative DKA is difficult because many symptoms of DKA are nonspecific; in addition, it is difficult to identify symptoms while the patient is under the influence of general anesthesia [11]. Furthermore, as in this case, DKA can occur even when the recommended withdrawal period is followed. Several proposals have been made for identifying and managing SGLT2i-associated DKA [17]. Among them, checking for the presence of ketones and BGA is primary. These measurements may be useful for the early detection of DKA and prevention of exacerbations.

The sodium nitroprusside-based test for urine “ketones” has a low threshold of detection for acetoacetate, is relatively insensitive to acetone, and fails to detect β-hydroxybutyrate [18]. Therefore, measurement of urinary ketones is not appropriate for early detection of complete DKA, and measurement of blood ketones using point-of-care-testing is preferred when possible.

This case was diagnosed with DKA intraoperatively. There was no preoperative acidosis, and ketosis was not considered. The possibility that preoperative ketosis was present cannot be ruled out. It is also possible that the addition of surgical stress may have led to DKA. Therefore, medical professionals should conduct perioperative measurements of blood ketone bodies and BGA.

## Conclusions

There are several ways to prevent SGLT2i-related eDKA in the perioperative period. Discontinuation of SGLT2i three to four days before surgery is one such way. However, it has been shown that intraoperative eDKA can occur even after empagliflozin has been discontinued for more than three days. Perioperative measurement of ketones and BGA, three days after discontinuation of SGLT2i, is useful for the prevention and early detection of intraoperative eDKA.
